# Laboratory Guide for the Bacteriologist

**Published:** 1895-09

**Authors:** 


LABORATORY GUIDE FOR THE BACTERIOLOGIST. By LANGDON FORTHINGHAM, M. D. V., Assistant in Bacteriology and Veterinary Science, Sheffield Scientific School, Yale University. Illustrated. Price, 75c. Philadelphia: W. B. Saunders, 925 Walnut street. 1895.

This small volume has been prepared for the convenience of the bacteriologist. It consists of a compilation of recognised methods and formulas of staining agents, used for the detection of the various forms of bacteria.

A few well executed illustrations are used in explanation of the text. E. W.

